# The Role of Microclot Formation in an Acute Subarachnoid Hemorrhage Model in the Rabbit

**DOI:** 10.1155/2014/161702

**Published:** 2014-07-07

**Authors:** Lukas Andereggen, Volker Neuschmelting, Michael von Gunten, Hans Rudolf Widmer, Javier Fandino, Serge Marbacher

**Affiliations:** ^1^Department of Neurosurgery, Bern University Hospital, Inselspital Bern, 3012 Bern, Switzerland; ^2^Laboratories for Neuroscience Research in Neurosurgery, Boston Children's Hospital, Boston, MA 02115, USA; ^3^Harvard Medical School, Boston, MA 02115, USA; ^4^Department of Intensive Care Medicine, Bern University Hospital, Inselspital Bern, 3012 Bern, Switzerland; ^5^Department of Neurosurgery, University Hospital Cologne, 50924 Cologne, Germany; ^6^Institute of Pathology Länggasse, 3012 Bern, Switzerland; ^7^Department of Neurosurgery, Kantonsspital Aarau, 5001 Aarau, Switzerland

## Abstract

*Background.* Microvascular dysfunction and microthrombi formation are believed to contribute to development of early brain injury (EBI) after aneurysmal subarachnoid hemorrhage (SAH). *Objective.* This study aimed to determine (i) extent of microthrombus formation and neuronal apoptosis in the brain parenchyma using a blood shunt SAH model in rabbits; (ii) correlation of structural changes in microvessels with EBI characteristics. *Methods.* Acute SAH was induced using a rabbit shunt cisterna magna model. Extent of microthrombosis was detected 24 h post-SAH (*n* = 8) by fibrinogen immunostaining, compared to controls (*n* = 4). We assessed apoptosis by terminal deoxynucleotidyl transferase nick end labeling (TUNEL) in cortex and hippocampus. *Results.* Our results showed significantly more TUNEL-positive cells (SAH: 115 ± 13; controls: 58 ± 10; *P* = 0.016) and fibrinogen-positive microthromboemboli (SAH: 9 ± 2; controls: 2 ± 1; *P* = 0.03) in the hippocampus after aneurysmal SAH. *Conclusions.* We found clear evidence of early microclot formation in a rabbit model of acute SAH. The extent of microthrombosis did not correlate with early apoptosis or CPP depletion after SAH; however, the total number of TUNEL positive cells in the cortex and the hippocampus significantly correlated with mean CPP reduction during the phase of maximum depletion after SAH induction. Both microthrombosis and neuronal apoptosis may contribute to EBI and subsequent DCI.

## 1. Introduction

Aneurysmal subarachnoid hemorrhage (SAH) is a devastating cerebrovascular disease with high mortality and disability rates [1]. Intensive research in recent years revealed many different causes of SAH, including cerebral vasospasm, early brain injury (EBI) mediated by impaired microcirculatory function, microthrombosis, cortical spreading depression, oxidative stress, inflammation, and apoptosis. All of these conditions cause delayed cerebral ischemia (DCI) and thereby influence clinical outcome [1–6]. However, the relationship of microthrombi formation to early brain injury and neuronal apoptosis after SAH still remains unclear. To model the physiological situation in humans with aneurysmal SAH [7], this study investigated the association between early injury after SAH, microclot formation, and apoptosis in an extra-ntracranial blood shunt model in the rabbit.

## 2. Materials and Methods

### 2.1. Study Design

The study was incorporated as a subproject of ongoing experimental studies and performed in accordance with the National Institutes of Health guidelines for the care and use of experimental animals and with the approval of the Animal Care Committee of the Canton of Bern, Switzerland (approval no. 107/09). Of 12 three-month-old female New Zealand rabbits weighing 3.3–4.6 kg, four animals served as sham-operated controls. In eight animals, experimental SAH was performed as described below. The animals were housed in groups (two to four animals per cage) at 22–24°C under a 12-hour light-dark cycle with unrestricted access to food and tap water. All surgical procedures were performed under sterile conditions at the Experimental Surgical Institute, Department of Clinical Research, Bern University Hospital, Bern, Switzerland. A veterinary anesthesiologist monitored the animals during surgery and throughout anesthetic recovery.

### 2.2. Anesthesia, Clinical Observation, and Sacrifice

Induction of general anesthesia was performed by subcutaneous administration of ketamine (30 mg/kg; Ketalar, 50 mg/mL, Pfizer, Zurich, Switzerland) and Xylazine (6 mg/kg; Xylapan 20 mg/mL, Vetoquinol, Bern, Switzerland) and continued intravenously. Humidified oxygen was provided to the spontaneously breathing animals. The animals underwent clinical observation during anesthetic recovery (first three hours) and from then on every six hours. Euthanasia was performed 24 hours post-SAH induction under the same anesthesia as previously described, by intra-arterial bolus injection of sodium thiopental (40 mg/kg) (Pentothal, Ospedalia AG, Hünenberg, Switzerland).

### 2.3. SAH Induction, Instruments, and Data Acquisition

The intracerebral pressure- (ICP-) controlled blood shunt model was used to induce SAH as described previously [7, 8]. Briefly, the cisterna magna was punctured with a pediatric spinal access needle (22 G × 40 mm) and connected via pressure tube and interposed three-way stopcock to the subclavian artery. The three-way stopcock was used for blood pressure measurement and to control bleeding. Neuromonitoring including an ICP monitor catheter tip (OLM Intracranial Pressure Monitoring Kit, Camino, Model 110-4B, Camino Laboratories, San Diego, CA, USA) and two laser-Doppler flowmetry fine needle probes (MNP110XP, 0.48 mm diameter, Oxford Optronix Ltd., Oxford, UK) were positioned in the olfactory bulb and bilateral frontal lobe according to outer skull landmarks [9]. Standard cardiovascular monitoring (mean arterial blood pressure (MABP), heart rate, electrocardiogram, end-tidal CO_2_, and SaO_2_) was performed at a sampling rate of 100 Hz (Datex S5 Monitor GE Medical Systems Switzerland, Glattbrugg, Switzerland), transferred via the analog output interface to an analog-digital converter/data logger, stored (Biopac MP100 and acknowledge version 3.8.1; BIOPAC Systems, Inc., Goleta, CA, USA), and processed for preanalysis using scripting software (Mathworks Inc, Natick, MA, USA). Pressures were zeroed at heart level before and after each session, and pressure calibration of the AD converter and data-logging system was done once before the series started.

SAH was initiated by opening the blood shunt to let blood stream into the atlantooccipital cistern under arterial pressure. After opening the shunt, ICP increased until it reached a plateau. If this plateau phase was maintained for more than 10 seconds, the shunt was closed. The shunt was also closed if ICP decrease occurred spontaneously (no later than 30 seconds from start of the plateau phase—we therefore did not allow for potential rebleeding). Control animals underwent frontal osteotomy with ICP and cerebral blood flow (CBF) monitoring placement, as well as puncture of the cisterna magna without blood shunting. MABP, ICP, and bilateral regional cerebral blood flow rCBF were recorded for 5 minutes before (baseline) and 20 minutes after initiation of SAH (steady state).

### 2.4. Tissue Processing, Histology, and Immunohistochemistry

**Figure 1:**
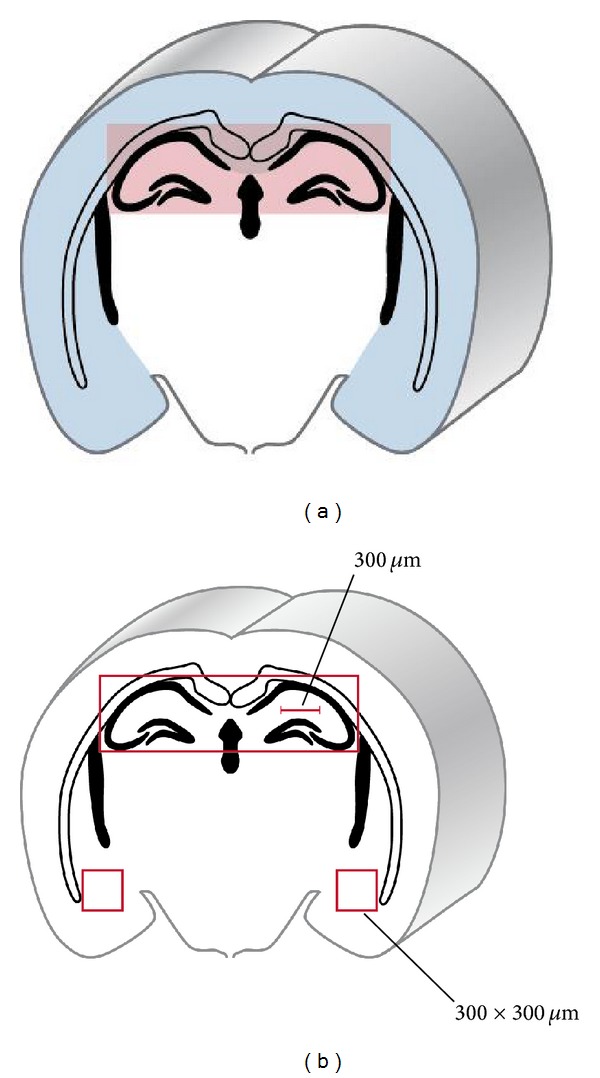
Microclot formation analyzed in the hippocampus and cortex. (a) Schematic drawing of regions used for analysis of fibrinogen immunostained microclots in the hippocampus (red bar) and the cortex (blue bar). (b) Specific regions of interest (ROI) from the hippocampus and cortex used for dimensional analysis.

Intracardiac perfusion-fixation was carried out 24 hours after SAH-induction at room temperature with 400 mL of 0.1 M phosphate-buffered solution (PBS) followed by 400 mL fixative (4% paraformaldehyde in 0.1 M PBS, pH 7.3). Brains were removed from the skull and cut into four blocks between the forebrain (olfactory bulb) and cerebellum, embedded in paraffin, and cut into consecutive 7 *μ*m sections. The cut surface of block one was placed through the cortical punch defect of the ICP and rCBF probes. The first section of blocks two to four was stained with hematoxylin and eosin, and the most representative fields containing the hippocampus and basal cortex were selected for additional cuts of ten consecutive sections used for immunohistochemical analysis in order to analyze the same subsection to eliminate bias. Apoptosis was detected using terminal deoxynucleotidyl transferase deoxyuridine triphosphate (dUTP) nick end labeling (TUNEL, Roche Diagnostics AG, Rotkreuz, Switzerland) as described above [7]. Quantitative analysis of apoptosis was performed within predefined regions of interest (ROI) of 300 *μ*m × 300 *μ*m on coronal sections for each hemisphere (Figure 1(b)). Thereby, 9 ROIs were used for analysis of apoptosis in the basal cortex (9 × 300 *μ*m × 300 *μ*m) and 3 ROIs along the hippocampal sectors CA1 and CA3 with (3 × 300 *μ*m × 300 *μ*m). Nuclei were counterstained with DAPI (Roche Diagnostics AG, Basel, Switzerland). Slides were visualized under a fluorescent microscope operating with a digital camera (Olympus BX 51, Olympus, Hamburg, Germany) using 2, 10, and 20x magnifications. Thereby, TUNEL red, FJB green, and DAPI blue were excited at 570–620 nm (maximum 580 nm), 450–490 nm (maximum 480 nm), and 340–380 nm (maximum 350 nm), respectively. The extent of microthrombosis was detected by fibrinogen immunohistochemistry using the Leica Bond III IHC staining system and analyzed in a blinded manner according to the schematic drawing depicted in Figure 1. For the fibrinogen immunohistochemistry, heat-induced epitope retrieval was carried out at 95°C for 20 minutes, followed by incubation with the primary antibody (polyclonal fibrinogen sheep anti-rabbit antibody; Acris, AP08879PU-N, 1:2′000, Herford, Germany) and secondary antibody (biotin-SP-conjugated AffiniPure donkey anti-sheep antibody; Jackson ImmunoResearch Laboratories, 713-065-003, 1:1′000, West Grove; USA), followed by incubation with a streptavidin-conjugated horseradish peroxidase reagent (Streptavidin-HRP, Leica Biosystems, RE7104).

### 2.5. Statistical Analysis

Data were analyzed and visualized using IBM SPSS statistical software Version 21.0 (IBM Corp., New York, NY, USA) and processed for preanalysis using Matlab scripting software (Mathworks Inc., Natick, MA, USA). Values were expressed as mean ± SEM. The differences between the normally distributed data of two groups were analyzed by Student's *t*-test and among three or more groups by one-way ANOVA, respectively, with Bonferroni post hoc testing. ANOVA regression analysis was used for calculation of correlations between effects of SAH on the CPP and the number of fibrinogen positive microvessels and the TUNEL-positive cells. The strength of linear correlations was expressed by the linear regression coefficient (reg coeff *r*) and its squared value *r* ^2^. A significance level of *P* < 0.05 was applied to all tests.

## 3. Results

### 3.1. Gross Examination of Brain and Pathophysiology

There was no mortality in this study. In general, the mortality rate is about 20%–30% due to respiratory arrest or severe bradycardia at the time of acute SAH [8]. There were no signs of cerebrospinal fluid leakage along the frontal osteotomy sites or at the site of nuchal cisterna magna puncture (data not shown). Twenty-four hours after SAH, rabbits (*n* = 8) demonstrated extensive coagulated diffuse subarachnoid blood in the chiasmatic, basal, prepontine cisterns, and cistern magna. No subarachnoid blood was observed in control animals (*n* = 4). All SAH animals demonstrated marked increases in ICP (6.2 ± 1.7 mmHg baseline versus 49.6 ± 11.9 mmHg peak; *P* < 0.001) with a corresponding decrease in bilateral rCBF (mean of both hemispheres: 36.3 ± 20.3% from baseline, *P* < 0.001) and the CPP (32.3 ± 15.0% of baseline, *P* < 0.001) within the first three minutes after induction of SAH. The ICP returned within 20 minutes to a steady state that was slightly higher than baseline but was not statistically different from baseline values (baseline: 6.2 ± 1.7 mmHg, steady state: 19.4 ± 4.3 mmHg; *P* = 0.051). Accordingly, both rCBF and CPP recovered to a state that was not significantly below baseline levels (mean of both hemispheres, rCBF: 76.8 ± 15.2% of baseline, *P* = 0.18; CPP: 81.2 ± 9.6% of baseline, *P* = 0.15). The mean arterial blood pressure remained unchanged throughout. A summary of pathophysiological characteristics is provided in Table 1.

### 3.2. Immunohistochemistry and Analyses

**Figure 2:**
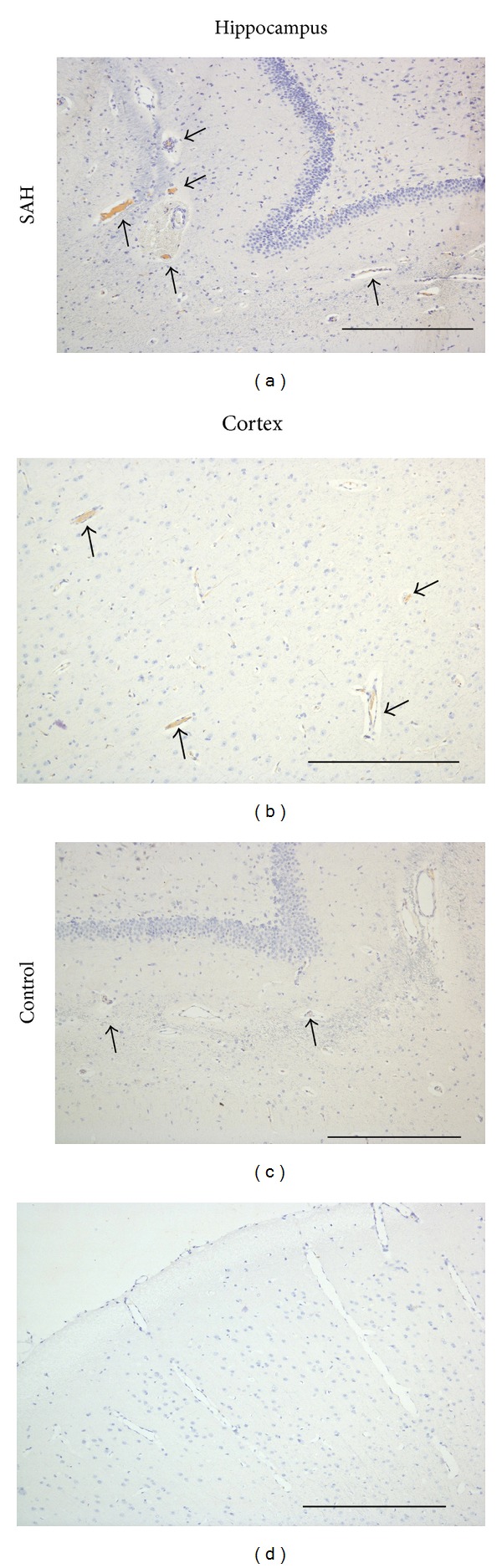
Microclot formation after induction of subarachnoid hemorrhage. Representative images showing fibrinogen positive vessels (brown staining; black arrows) in the hippocampus ((a), (c)) and cortex ((b), (d)) after SAH ((a), (b)) and in controls ((c), (d)). Scale bars = 400 *μ*m.

**Figure 3:**
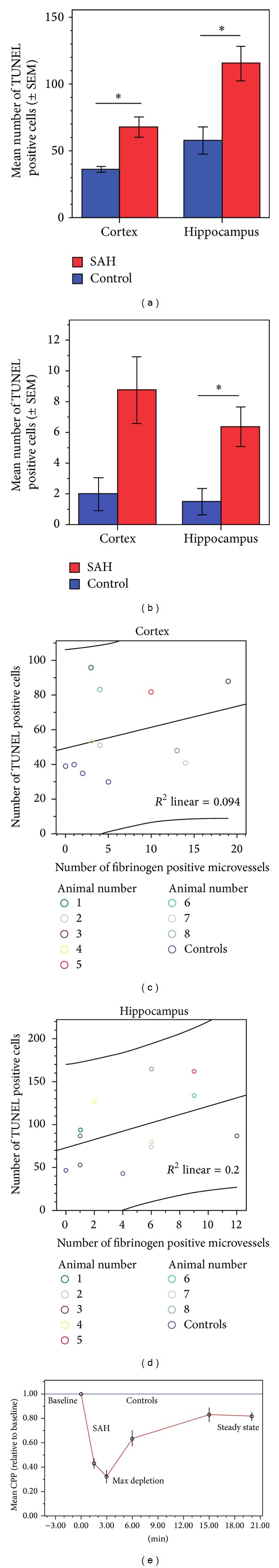
Evidence of microclot formation, neuronal apoptosis, and CPP depletion after experimental SAH. Quantification of TUNEL-positive cells and fibrinogen-positive stained microthrombi in cortex and hippocampus. (a) There were significant increases in the number of TUNEL-positive cells in both cerebral cortex and hippocampus as compared to controls (*P* = 0.016 and *P* = 0.017, resp., Student's *t*-test). (b) There was a nonstatistically significant trend towards an increase in fibrinogen-positive microvessels in the cerebral cortex (*P* = 0.06) and a statistically significant increase in fibrinogen-positive microvessels in the hippocampus (*P* = 0.03). No correlations of TUNEL-positivity with fibrinogen-positivity were observed in cortex (*R* ^2^ = 0.094; (c)) or in hippocampus (*R* ^2^ = 0.2; (d)). All data are expressed as mean ± SEM, *n* = 8 in SAH group, and *n* = 4 in control group. A *P* value of <0.05 was considered statistically significant. (e) Time course of the CPP depletion after induction of SAH compared to control animals.

Fibrinogen staining showed distinct microclot formation in vessels of the hippocampus (Figures 2(a) and 2(c)) and cerebral cortex (Figures 2(b) and 2(d)) after SAH ((a), (b)) compared to control animals ((c), (d)). Immunohistochemistry analysis revealed a significant increase of the number of TUNEL-positive cells in both cerebral cortex and hippocampus (Figure 3(a)). Namely, there were 68 ± 8 TUNEL-positive cells in the cortex after SAH compared to 36 ± 2 cells in the control animals (differences between means 32 ± 11; *P* = 0.017). In the hippocampus, there were 115 ± 13 TUNEL-positive cells after SAH compared to 58 ± 10 positive cells in the control animals (differences between means 58 ± 20; *P* = 0.016). Taking into account the differences in the density of neurons in the hippocampus and the cerebral cortex, immunohistochemistry analyses showed in the hippocampus 115 ± 13 TUNEL-positive cells after SAH compared to 68 ± 8 positive cells in the cerebral cortex (differences between means 48 ± 15; *P* = 0.014). In control animals, there were 58 ± 10 TUNEL-positive cells in the hippocampus compared to 36 ± 2 in the cerebral cortex (differences between means 22 ± 9; *P* = 0.13).

A tendency towards increased mean number of fibrinogen-positive microvessels in cerebral cortex was noted (9 ± 2 fibrinogen positive cells after SAH compared to 2 ± 1 positive cells in control animals; *P* = 0.06). There was a significant increase in fibrinogen-positive microvessels in the hippocampus (9 ± 2 for SAH compared to 2 ± 1 in sham controls, *P* = 0.03) (Figure 3(b)).

### 3.3. Correlation between Apoptosis and Microclot Formation

There was no correlation between microclot formation (number of fibrinogen positive clots) and apoptosis (number of TUNEL positive cells) in the cerebral cortex (reg coeff *r* = 0.31, *r* ^2^ = 0.094, *P* = 0.3; Figure 3(c)) or the hippocampal region (reg coeff *r* = 0.45, *r* ^2^ = 0.2, *P* = 0.14; Figure 3(d)).

### 3.4. Correlation between CPP Depletion and Apoptosis Respectively Microclot Formation

**Figure 4:**
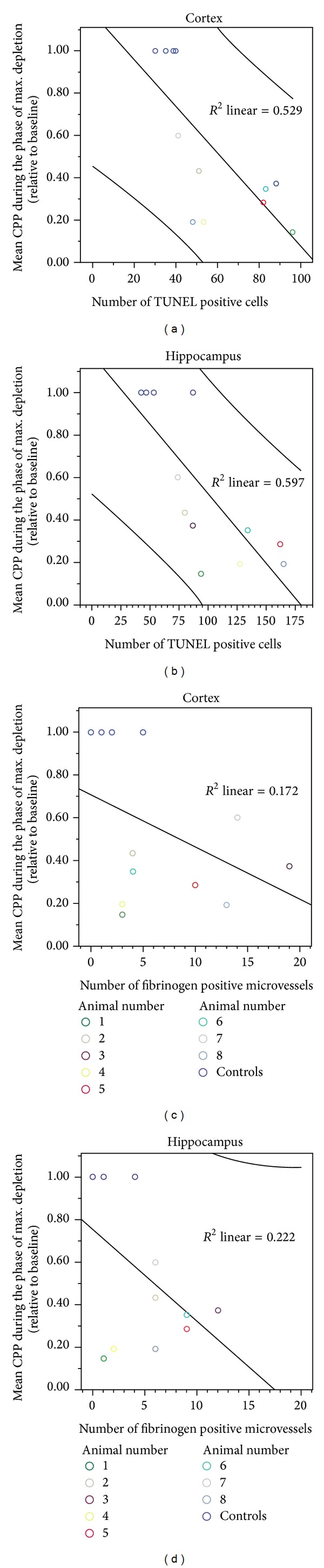
In the SAH group, a significant reduction in CPP was observed within the first three minutes of the phase of maximum depletion compared to the baseline or the steady state (*P* < 0.001). A significant positive linear correlation between the mean CPP during the phase of maximum depletion after induced SAH and the total number of TUNEL positive cells was found in both the cortex (*r* ^2^ = 0.53, *P* = 0.007; (a)) and the hippocampus (*r* ^2^ = 0.60, *P* = 0.003; (b)). There was no correlation between CPP depletion and fibrinogen positive microvessels in either cortex (*r* ^2^ = 0.17, (c)) or hippocampus (*r* ^2^ = 0.22, (d)). Graphs include the 95% confidence intervals.

CPP showed maximal depletion within the first 3 minutes after induction of SAH (Figure 3(e)). A significant linear correlation was observed between CPP reduction within the first three minutes after SAH and the total number of TUNEL positive cells in the cortex (reg coeff *r* = 0.73, *r* ^2^ = 0.53, *P* = 0.007; Figure 4(a)) as well as in the hippocampus (reg coeff *r* = 0.77, *r* ^2^ = 0.60, *P* = 0.003; Figure 4(b)). However, no significant correlation was detected between relative CPP depletion within the first three minutes and the number of fibrinogen stained microvessels in the cortex (reg coeff *r* = 0.42, *r* ^2^ = 0.17, *P* = 0.18; Figure 4(c)) or in the hippocampus (reg coeff *r* = 0.47, *r* ^2^ = 0.22, *P* = 0.12; Figure 4(d)).

## 4. Discussion

### 4.1. Animal Model

In recent years, evidence has indicated that EBI and DCI largely contribute to the unfavorable outcome and mortality after aneurysmal SAH [1, 2]. Parenchymal apoptosis and microthrombosis after aneurysmal SAH are considered to be mainly involved in EBI and contributing to DCI [10–12]. Although different animal models of SAH exist [9, 13–15], it is important to investigate the impact of microthrombosis and apoptosis on EBI in animal models that represent acute pathophysiological features of SAH such as the endovascular perforation models [16–19] or ICP controlled blood prechiasmatic injection [20].

The potential important advantage of using a rabbit aneurysmal SAH model to investigate microthrombosis formation postaneurysmal SAH is the fact that the rabbit coagulation system is very similar to that in humans [21, 22]. Human-resemblance of larger animals makes them an attractive tool to provide new insights into the study of microvascular thrombosis [23, 24].

Furthermore, following aneurysm rupture, there is a rapid increase in intracranial pressure and consequent decrease in cerebral perfusion pressure [25]. Cerebral ischemia caused by increased intracranial pressure and reduced cerebral blood flow induces severe injury to the brain tissue and cerebral microvasculature [26]. To take advantage of the ability to control for ICP increase to investigate the effect on the mechanisms of cell apoptosis and intraparenchymal microclot formation, our rabbit model closely mimics these human pathophysiological features of aneurysm rupture by arterial blood-inflow into a closed cranium [7, 8].

### 4.2. Microclot Formation

Recent studies detected microclots following aneurysmal SAH in humans and in models such as mice and rats [5, 19, 27–31]. In a rat model of endovascular perforation, platelet aggregates were detected in the cerebral pial microvasculature as early as 10 minutes after SAH, reaching a peak at 24 hours, and were undetectable at 48 hours [32, 33]. For the intraparenchymal microcirculation, platelets have been shown to aggregate in parenchyma microvessels within 10 minutes after SAH and persist for up to 24 hours [34].

It is still unknown whether parenchymal microvessels respond the same way as pial vessels [18, 35]. In our rabbit blood shunt model, the hippocampal brain parenchyma showed a selective vulnerability to SAH induced microthrombosis formation within 24 hours. Compared to the mouse perichiasmatic injection model used by Sabri et al. [29, 36], they observed a significant increase of microthrombosis in both the hippocampus and cerebral cortices. Although brain injury due to aneurysmal SAH causes global parenchymal damage [37, 38], vulnerability to subcortical brain regions may differ [39]. This might explain the SAH-induced significant increase of microthrombosis in the rabbit brain parenchyma at the hippocampal levels but not the cortex. Furthermore, the number of animals used in our study is small, which might explain missing correlation data in the cortex.

Finally, different mechanisms may contribute to microclot formation. An experimental model of SAH in the rat showed that a hypercoagulable state occurs immediately after injury. This abnormality in coagulation profile seemed to be a response mechanism for the acute traumatic events caused by induction of SAH in rats and may predispose them to microthrombus formation [40]. In humans, elevation of platelet activating factor and coagulation factors after SAH has been described [41]. Supporting the idea of microclot formation, markers of hypercoagulation and platelet activation increase dominantly in CSF and jugular blood compared to systemic levels after aneurysmal SAH, indicating a cerebral origin [42]. To sum up, activation of the coagulation cascade, impaired fibrinolytic activity, and inflammatory processes are particularly regarded as possible mechanisms for microclot formation [5], and different coagulation profiles among various species might be considered for future investigations regarding microclot formations.

### 4.3. Brain Apoptosis

Although the exact mechanism of intravascular coagulation after aneurysmal SAH is unknown, microclot formation may contribute to decreased cerebral blood flow, subsequent ischemic injury, and neuronal apoptosis supporting the development of DCI [32]. It is still a matter of debate whether the blood clot itself or the transient global ischemia after increase of the intracranial pressure is responsible for the microcirculatory changes after SAH [36]. In our rabbit model, 24 hours after SAH-induction, microclot formation paralleled by apoptotic brain cells marked by TUNEL was predominantly distributed in the hippocampus, a brain region known to be particularly vulnerable to transient ischemia [43]. This finding is consistent with several autopsy studies that detected microclots in small parenchymal vessels and demonstrated a correlation between these microclot densities and the location and severity of histological evidence of ischemia [27]. It is well know that a brief period of global brain ischemia mainly causes cell death in hippocampal subfields neurons in rodents and humans, whereas other neurons are much less vulnerable [44, 45]. Furthermore, microclot formation and neuronal apoptosis were detected in both hemispheres, indicating a more global disease after SAH than the effect of local ischemia caused by microclot formation. In MRI studies in SAH patients, delayed ischemic lesions were observed bilaterally, regardless of the site of aneurysm rupture or vasospasm [46, 47]. Similarly, microembolic signals detected with transcranial Doppler (TCD) in 40 patients with aneurysmal SAH were mostly noted bilaterally and were associated with the development of cerebral ischemic symptoms and not related to vasospasm [31]. In an endovascular perforation model in mice, positive antithrombin staining was detected bilaterally in a scattered distribution with a peak at 48 hours and then decreased gradually [30]. However, the disadvantage of this murine model using endovascular perforation is the uncertainty to determine if rebleeding occurred over the study time.

### 4.4. Correlation of Microthrombosis, Neuronal Injury, and CPP Depletion

CPP shortage during the hyperacute phase of SAH significantly correlated with the degree of apoptosis and neurodegeneration in the hippocampus and cortex 24 hours after experimental SAH. However, no significant correlation was found between CPP depletion and the number of fibrinogen positive microvessels in either the cortex or the hippocampus. Furthermore, there was no significant correlation between structural changes in microvessels due to early brain injury comparing TUNEL-positivity neither in the cortex nor in the hippocampus in our study findings. This is in contrast to the studies of Sabri et al. [29, 36], where there were significant correlations of microthrombosis formation and neuronal apoptosis. One possible explanation might be that an inflammatory response that accompanies neural injury has both positive and negative effects [48]. There is extensive cross talk between inflammation and coagulation, whereby not only does inflammation lead to activation of coagulation but coagulation also considerably affects inflammatory activity [49]. It is known that, within a few hours after neuronal injury to the central nervous system, numerous neutrophils induce an inflammatory reaction and express high levels of the atypical growth factor oncomodulin, a crucial factor for neuronal regeneration, and cell survival [50]. Inflammation may also cause neuroprotection via inhibition of apoptosis after ischemia [51]. However, the exact effects and mechanisms of inflammation, microthrombosis, apoptosis, and neuroprotection in the context of aneurysmal SAH remain unknown and their temporal interactions need to be analyzed in further detail.

To sum up, microthrombosis in the cerebral cortex and hippocampus in a rabbit model of SAH is an important finding by itself. To the best of our knowledge, this is the first time that early microclot formation has been demonstrated in a rabbit model of SAH. Because rabbits have a coagulation cascade very similar to that seen in humans [21, 22], these findings might provide a further piece of the puzzle in our understanding of pathophysiological aspects taking place in mammals after SAH and might contribute to successful translational studies in human trials.

## 5. Conclusion

This study found evidence of microclot formation and neuronal apoptosis after experimental SAH in a rabbit blood shunt model. Both microthrombosis and neuronal apoptosis may contribute to EBI and subsequent DCI in a distinct pathway. Long-term survival studies are mandatory to further analyze the impact and temporal characteristics—as well as the chronological sequence—of microthrombosis and apoptosis impact on DCI.

## Figures and Tables

**Table 1:** Pathophysiological characteristics of SAH animals (*n* = 8)^a^.

Time point	MABP (mmHg)	ICP (mmHg)	Relative CPP (% of BL)	rCBF of both hemispheres (% of BL)
Baseline	68.4 ± 6.1	6.2 ± 1.7	100	100
Peak	69.5 ± 7.8	49.6 ± 11.9^∗†^	32.3 ± 15.0^∗†^	36.3 ± 20.3^∗†^
Steady state	70.3 ± 5.3	19.4 ± 4.3	81.2 ± 9.6	76.8 ± 15.2

Abbreviations: BL = baseline; CPP = cerebral perfusion pressure; ICP = intracranial pressure; MABP = mean arterial blood pressure; rCBF = regional cerebral blood flow.

^a^Values are expressed as mean ± SD.

∗Significantly different compared to baseline values (*P* < 0.001).

^†^Significantly different compared to steady-state values (*P* < 0.001).
